# Effects of Arsenic on Osteoblast Differentiation *in Vitro* and on Bone Mineral Density and Microstructure in Rats

**DOI:** 10.1289/ehp.1307832

**Published:** 2014-02-14

**Authors:** Cheng-Tien Wu, Tung-Ying Lu, Ding-Cheng Chan, Keh-Sung Tsai, Rong-Sen Yang, Shing-Hwa Liu

**Affiliations:** 1 Institute of Toxicology,; 2 Department of Geriatrics and Gerontology,; 3 Department of Laboratory Medicine, and; 4 Department of Orthopaedics, College of Medicine, National Taiwan University, Taipei, Taiwan; 5 Department of Medical Research, China Medical University Hospital, China Medical University, Taichung, Taiwan; * These authors contributed equally to this study.

## Abstract

Background: Arsenic is a ubiquitous toxic element and is known to contaminate drinking water in many countries. Several epidemiological studies have shown that arsenic exposure augments the risk of bone disorders. However, the detailed effect and mechanism of inorganic arsenic on osteoblast differentiation of bone marrow stromal cells and bone loss still remain unclear.

Objectives: We investigated the effects and mechanism of arsenic on osteoblast differentiation *in vitro* and evaluated bone mineral density (BMD) and bone microstructure in rats at doses relevant to human exposure from drinking water.

Methods: We used a cell model of rat primary bone marrow stromal cells (BMSCs) and a rat model of long-term exposure with arsenic-contaminated drinking water, and determined bone microstructure and BMD in rats by microcomputed tomography (μCT).

Results: We observed significant attenuation of osteoblast differentiation after exposure of BMSCs to arsenic trioxide (0.5 or 1 μM). After arsenic treatment during differentiation, expression of runt-related transcription factor-2 (Runx2), bone morphogenetic protein-2 (BMP-2), and osteocalcin in BMSCs was inhibited and phosphorylation of enhanced extracellular signal-regulated kinase (ERK) was increased. These altered differentiation-related molecules could be reversed by the ERK inhibitor PD98059. Exposure of rats to arsenic trioxide (0.05 or 0.5 ppm) in drinking water for 12 weeks altered BMD and microstructure, decreased Runx2 expression, and increased ERK phosphorylation in bones. In BMSCs isolated from arsenic-treated rats, osteoblast differentiation was inhibited.

Conclusions: Our results suggest that arsenic is capable of inhibiting osteoblast differentiation of BMSCs via an ERK-dependent signaling pathway and thus increasing bone loss.

Citation: Wu CT, Lu TY, Chan DC, Tsai KS, Yang RS, Liu SH. 2014. Effects of arsenic on osteoblast differentiation *in vitro* and on bone mineral density and microstructure in rats. Environ Health Perspect 122:559–565; http://dx.doi.org/10.1289/ehp.1307832

## Introduction

Environmental arsenic pollution causes a significant global problem for human health. Arsenic in the environment contaminates soil and groundwater and is released to food and drinking water. In certain areas of the world where arsenic contamination is endemic, such as Bangladesh, China, India, Mexico, Romania, Taiwan, and others, arsenic-related disease is prevalent as a result of drinking arsenic-contaminated water (Garelick et al. 2008). Arsenic exposure has been associated with increased incidence of various health conditions such as hypertension, cardiovascular disorders, skin lesions, cancer, and spontaneous pregnancy loss (Abhyankar et al. 2012; Bloom et al. 2010; Chen et al. 2009). In areas with high levels of arsenic contamination in drinking water, increased mortality has been reported for males and females with several cancers, including bone cancer, compared with the local reference population (Tsai et al. 1999). Arsenic is known to replace phosphorus and localize in the bone, where it may remain for years. Feussner et al. (1979) observed bone marrow abnormality in a patient with severe arsenic poisoning. Some epidemiological studies have reported that arsenic exposure augments the risk of bone disorders (Akbal et al. 2013; Haag et al. 1974; Lever 2002). In a recent study, Akbal et al. (2013) found that arsenic exposure in male participants was associated with bone metabolism, suggesting that arsenic exposure may be a possible cause of osteopenia. Hu et al. (2012) observed that short-term exposure of high-dose inorganic arsenic (10 mg/kg/day) to rats through an unusual route of arsenic exposure (intraperitoneal injection) affected bone remodeling. However, the mechanism of arsenic on the bone system are still unclear.

Arsenic exposure induces complex modes of action to disturb physiological functions (Abhyankar et al. 2012; Bailey et al. 2013). Arsenic stress could lead to activation of cellular and molecular signal transduction in target organs (Qian et al. 2003; Wang et al. 2012). Extracellular signal-regulated kinase (ERK), a member of mitogen-activated protein kinases (MAPK), has been found to contribute to arsenic-induced toxicological responses (Bonati et al. 2006; Ivanov and Hei 2013; Wang et al. 2013). ERK activation also plays an important role in osteoblast differentiation and osteoclast formation (Lai et al. 2001; Matsushita et al. 2009). In addition, ERK can regulate the expression of osteoblast differentiation-related signaling molecules, such as runt-related transcription factor 2 (Runx2), bone morphogenetic protein-2 (BMP-2), and core-binding factor a1 (Celil and Campbell 2005; Wu et al. 2012). However, the effect of arsenic on ERK signaling during osteoblast differentiation still remains unclear. In the present study, we hypothesized that low-dose inorganic arsenic disturbs osteoblast differentiation from bone marrow stromal cells (BMSCs) through an ERK signaling pathway and induces bone loss. Our results showed that low-dose inorganic arsenic significantly decreased osteoblast differentiation from BMSCs via an ERK-dependent pathway *in vitro* and *in vivo*.

## Materials and Methods

*Animal experiments*. The Animal Research Committee of the College of Medicine, National Taiwan University, approved and conducted the study in accordance with the guidelines for the care and use of laboratory animals. A total of 32 male Wistar rats (6–8 weeks of age) were purchased from BioLASCO (Taipei, Taiwan). Two rats were housed per standard rat microisolator cage on aspen chip bedding in an animal room maintained at 22 ± 2°C with a 12-hr light/dark cycle. The animals were treated humanely and with regard for alleviation of suffering. Rats were provided standard chow diet (LabDiet #5053; LabDiet, St. Louis, MO, USA) and deionized, sterile water *ad libitum*. The maximum contaminant level of arsenic in drinking water in Taiwan is 0.01 ppm. For *in vivo* experiments, rats were randomly divided into three groups (8 animals/group), with each group receiving 0, 0.05, or 0.5 ppm As_2_O_3_ (arsenic trioxide; Sigma-Aldrich, St. Louis, MO, USA) in drinking water for 12 weeks.

After 12 weeks of arsenic exposure, 4 animals from each exposure group were sacrificed and the left and right tibias were removed. Left tibias were fixed in phosphate-buffered saline (PBS) containing 4% paraformaldehyde for 48 hr; BMD analysis was then performed by microcomputer tomography (μCT). Right tibias were decalcified with 10% sodium EDTA solution at 4^o^C for 1 month. The samples were then embedded in paraffin and sectioned to a thickness of 4 μm for immunofluorescence staining. The tibias and femurs from the remaining 4 animals/group were used to prepare BMSCs (Liu et al. 2011).

*Bone marrow cells*. Primary BMSCs were isolated from rats and cultured with or without the differentiation medium, as previously described (Çelebi et al. 2010). Briefly, BMSCs were prepared by removing tibias and femurs from rats under anesthesia (sodium pentobarbital; Sigma-Aldrich) and flushing the bone marrow cavity with growth medium (α-minimum essential medium; αMEM) supplemented with 10% fetal bovine serum (FBS), 100 units/mL penicillin, and 100 mg/mL streptomycin (all from Life Technologies, Carlsbad, CA, USA). Cells were then cultured in growth medium at 37^o^C in a humidified atmosphere of 5% CO_2_ in air. After 1 week of cell expansion, the adherent cells were treated with differentiation inducers (10^–8^ M dexamethasone, 10 μM β-glycerophosphate, and 50 μg/mL ascorbic acid; all from Sigma-Aldrich) in the medium to induce osteoblast differentiation.

*Cell viability assay*. BMSCs (2.5 × 10^4^/well) isolated from control rats were seeded in 24-well plates for 24 hr and then refreshed by the addition of growth medium. Cells were treated with 0–15 μM As_2_O_3_ for 24 hr (cultured a total of 48 hr) or with 0, 0.5, or 1 μM As_2_O_3_ for 3–18 days. Cell viability was measured by the MTT [3-(4,5-dimethyl-2-thiazolyl)-2,5-diphenyl-2H-tetrazolium bromide; Sigma-Aldrich] assay.

*Alkaline phosphatase (ALP) activity assay*. We examined ALP activity using an ALP activity assay kit (Alkaline Phosphatase liquicolor; Human Gesellschaft für Biochemica und Diagnostica mbH, Wiesbaden, Germany) following the manufacturer’s instructions. Briefly, BMSCs (2.5 × 10^4^/well) isolated from control or As_2_O_3_-treated rats were treated with 0, 0.5, or 1 μM As_2_O_3_, with or without 20 μM PD98059, for 7 days in differentiation medium. The medium was changed every 3 days. Cells were harvested using RIPA buffer and centrifuged at 13,000 × *g* for 30 min. We measured ALP activity in the supernatant; absorbance was read at 420 nm. Each sample was normalized by protein level.

*Calcium measurement*. To detect calcium concentrations in culture medium, we used a calcium concentration assay kit (*o*-cresolphthalein complexone kit; Teco Diagnostics, Anaheim, CA, USA) following the manufacturer’s instructions. Briefly, BMSCs (5 × 10^5^ cells/plate) isolated from control rats were cultured in differentiation medium with 0, 0.5, or 1 μM As_2_O_3_ for 5 or 14 days; the medium was changed every 3 days. Fifty microliters of culture media collected at the end of day 5 or day 14 was mixed with working reagent (*o*-cresolphthalein complexone) and calcium buffer for 2–3 min at room temperature. The absorbance was detected at 570 nm and the concentration calculated by the standard curve.

*Mineralized nodule formation assay*. Mineralization was detected by Alizarin red S staining. Briefly, BMSCs (2.5 × 10^4^/well) isolated from control or As_2_O_3_-treated rats were cultured in differentiation medium with 0, 0.5, or 1 μM As_2_O_3_, with or without 20 μM PD98059, for 20 days. The medium was changed every 3 days. Cells were washed with PBS buffer, fixed in ice-cold 75% (vol/vol) ethanol, and then stained with 2% (wt/vol) Alizarin red S (Sigma-Aldrich). The stained cells were incubated with 10% (wt/vol) cetylpyridinium chloride (Sigma-Aldrich) to elute the Alizarin red S, and the solution was collected from the cells and measured at an absorbance at 550 nm.

*Real-time reverse transcription polymerase chain reaction (RT-PCR).* BMSCs (2 × 10^5^/well) isolated from control rats were seeded in 6-well plates and treated with As_2_O_3_ at 0, 0.5, or 1 μM, with or without 20 μM PD98059, for 5–14 days. Every 3 days, the medium was replaced with differentiation medium. Cells were lysed and the total RNA was extracted using a kit (TRIzol; Life Technologies, Carlsbad, CA, USA). We determined relative mRNA expression by real-time quantitative PCR, as previously described (Hsu et al. 2013). Briefly, total RNA (0.5–1 μg) was used for reverse transcription of RNA to cDNA using avian myeloblastosis virus reverse transcriptase. Each sample (2 μL cDNA) was tested with real-time SYBR Green PCR reagent (Life Technologies) with specific primers: *18S* (forward: AGTC​CCTG​CCCT​TTGT​ACACA; reverse: CGAT​CCGA​GGGC​CTCA​CTA), *GAPDH* (forward: TGGC​ACAG​TCAA​GGCT​GAGA; reverse: CTTC​TGAG​TGGC​AGTG​ATGG), bone morphogenetic protein 2 (*Bmp2*) (forward: AAGC​CATC​GAGG​AACT​TTCA​GA; reverse: TCAC​AGGA​AATT​TTGA​GCTG​GC), and osteocalcin (OCN): (forward: TCTG​ACAA​AGCC​TTCA​TGTC​CA; reverse: ​AACG​GTGG​TGCC​ATAG​AT). Amplification was performed using an ABI StepOnePlus sequence detection system and StepOne 2.1 software (Applied Biosystems, Foster City, CA, USA).

*Western blotting*. Western blotting of proteins from BMSCs was performed as described previously (Wu et al. 2011). BMSCs were treated with As_2_O_3_ at 0, 0.5, or 1 μM, with or without 20 μM PD98059, for 6 hr or 7 days. Total protein (30–50 μg per sample) was subjected to electrophoresis on 8–10% SDS-polyacrylamide gels. The proteins were transferred to a polyvinylidene difluoride (PVDF) membrane and blocked with 5% fat-free milk in Tris-buffered saline/Tween–20 (TBST) buffer (20 mM Tris, 150 mM NaCl, 0.01% Tween–20, pH 7.5) for 1 hr. PVDF membranes were then incubated overnight at 4°C with primary antibody [ERK1/2, phosphorylated ERK1/2, Runx2, or GAPDH (Santa Cruz Biotechnology, Santa Cruz, CA, USA)] in BSA-TBST buffer. After washing in PBS and 0.01% Tween–20, the membranes were incubated with horseradish peroxidase–conjugated secondary antibody for 1 hr. The antibody-reactive bands were identified by enhanced chemiluminescence reagent (Millipore, Billerica, MA, USA) and exposed on Kodak radiographic film. The relative values of protein samples were normalized by the internal control GAPDH.

μ*CT evaluation of trabecular and cortical bones.* We assessed BMD in tibias by μCT scanning, as described previously (Takahata et al. 2012). Briefly, bones were scanned using μCT (Skyscan 1176; Bruker-MicroCT, Kontich, Belgium) with isotropic high resolution. Tibias were scanned at 80 keV and 309 μA with an aluminum plus copper filter, and the images were collected. Quantification of trabecular and cortical bone morphometric indices was performed in the regions of metaphysis and diaphysis in the proximal tibias, respectively. The trabecular/cortical BMD, trabecular bone volume fraction [bone volume/total volume (BV/TV)], trabecular/cortical thickness, and cortical area were measured and analyzed by Skyscan CTAn v.1.1.7 software (Bruker-MicroCT).

*Immunofluorescence staining*. The 4-μm sections of paraffin-embedded tibia were deparaffinized with xylene and washed with 90%, 75%, and 50% alcohol for 5 min each. Sections were then treated with 3% hydrogen peroxide–methanol solution to eliminate endogenous peroxidase activity and incubated with protease type XIV (0.5 mg/mL; Sigma-Aldrich) for 10 min. Tibia sections were blocked with 5% goat serum for 1 hr to prevent nonspecific binding, incubated overnight with the antibody for either Runx2 or phosphorylated ERK (1:200), and then treated with anti-rabbit or anti-mouse FITC (fluorescein isothiocyanate)–labeled secondary antibody (1:500; Sigma-Aldrich) for 1 hr. Finally, the sections were counterstained with Hoechst 33258 (1 μg/mL; Sigma-Aldrich).

*Statistical analysis*. Statistical analyses were performed using SPSS-16.0 software (IBM SPSS Statistics, Armonk, NY, USA). Data are expressed as mean ± SD. Data were analyzed for statistical significance using one-way analysis of variance followed by Holm-Sidak post analysis to test for differences between groups; *p* ≤ 0.05 was considered statistically significant.

## Results

**Figure 1:**
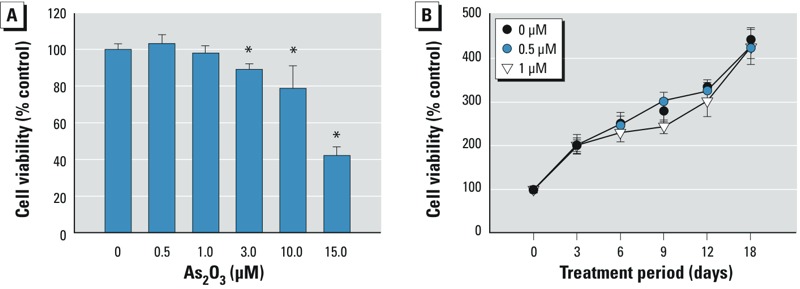
Effects of As_2_O_3_ on cell viability in BMSCs isolated from bones of control rats and cultured with 0–15 μM As_2_O_3_ in growth medium for 48 hr (*A*) or with 0, 0.5, or 1 μM As_2_O_3_ in differentiation medium for 3–18 days (*B*). Cell viability was determined by the MTT assay. Data are presented as mean ± SD for three independent experiments in triplicate. *p < 0.05, compared with the 0‑μM As_2_O_3_ group.

**Figure 2:**
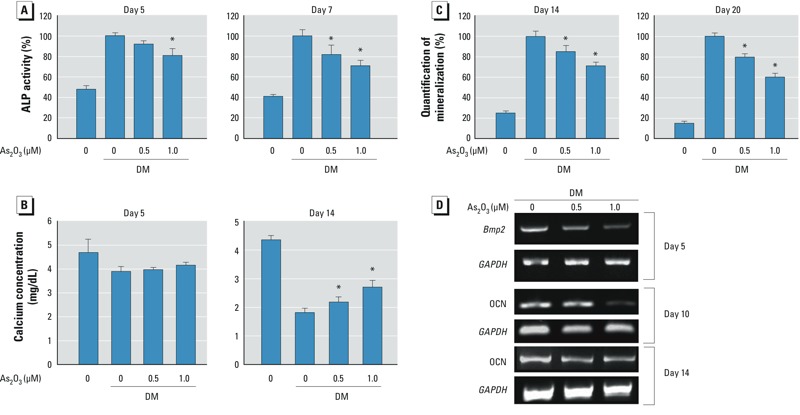
As_2_O_3_ reduced osteoblast differentiation of BMSCs. BMSCs were isolated from bones of control rats and cultured in differentiation medium (DM) with 0, 0.5, or 1 μM As_2_O_3_ for 5–20 days. ALP activity on days 5 and 7 (*A*), calcium absorption on days 5 and 14 (*B*), and osteoblast mineralization on days 14 and 20 (*C*) during differentiation. Data are presented as mean ± SD of three independent experiments in triplicate. (*D*) mRNA expression of *Bmp2* and osteocalcin (OCN) on days 5, 10, and 14 detected by real-time PCR. *GAPDH* was used as a control; results are representative of at least three independent experiments in triplicate. *n* = 3/group/day. *p < 0.05, compared with the 0‑μM As_2_O_3_ + DM group.

*Low-dose arsenic altered osteoblastogenesis from BMSCs*. As shown in Figure 1A, BMSCs treated with As_2_O_3_ at 3–15 μM for 48 hr showed decreased viability, but BMSCs treated with lower doses of As_2_O_3_ (0.5 and 1 μM) for 3–18 days showed no change in cell viability (Figure 1B). ALP was significantly decreased in BMSCs treated with 1 μM As_2_O_3_ at day 5 and in those treated with 0.5 or 1 μM As_2_O_3_ at day 7 (Figure 2A). We observed a decrease in calcium absorption in BMSCs treated with 0.5 or 1 μM As_2_O_3_ at 14 days but not at 5 days (Figure 2B). A decrease in osteoblast mineralization occurred in BMSCs treated with 0.5 or 1 μM As_2_O_3_ at days 14 and 20 (Figure 2C). We also observed mRNA expression of the osteoblastogenic markers BMP-2 and osteocalcin *Bmp2* was decreased by 0.5 or 1 μM As_2_O_3_ at day 5, and osteocalcin was decreased by 1 μM As_2_O_3_ at days 10 and 14 (Figure 2D). These results suggest that nontoxic low-dose As_2_O_3_ is capable of attenuating osteoblast differentiation of BMSCs.

**Figure 3:**
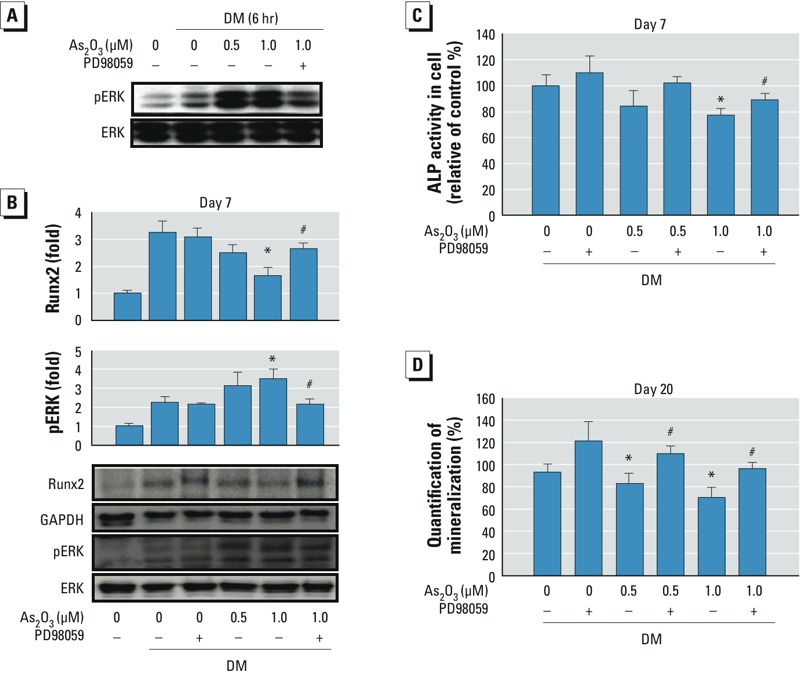
As_2_O_3_ enhanced ERK phosphorylation during osteoblast differentiation. BMSCs isolated from bones of control rats were cultured in differentiation medium (DM) with 0, 0.5, or 1 μM As_2_O_3_ in the presence (+) or absence (–) of the ERK inhibitor PD98059 (20 μM) for 6 hr to 20 days. ERK phosphorylation (*A*) and Runx2 expression (*B*) determined by Western blotting. (*C*) ALP activity. (*D*) Osteoblast mineralization. Data are presented as mean ± SD of four independent experiments. *p < 0.05, compared with the 0‑μM As_2_O_3_ + DM group. ^#^*p* < 0.05, compared with the respective As_2_O_3_ group without PD98059.

**Figure 4:**
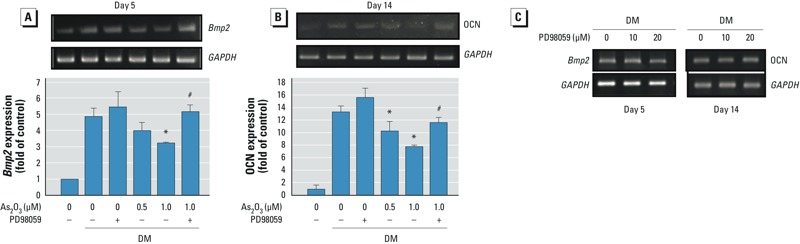
ERK inhibitor reversed effects of As_2_O_3_ on *Bmp2* (*A*) and OCN (*B*) mRNA expression during osteoblast differentiation in BMSCs isolated from bones of control rats. BMSCs were cultured in differentiation medium (DM) with 0, 0.5, or 1 μM As_2_O_3_ in the presence (+) or absence (–) of the ERK inhibitor PD98059 (20 μM) for 5–14 days. mRNA expression was determined by real-time PCR. Data are presented as mean ± SD of three independent experiments in triplicate. (*C*) Effect of PD98059 (10 and 20 μM) alone on gene expression of *Bmp2 *and OCN. Results are representative of at least three independent experiments. *p < 0.05, compared with the 0‑μM As_2_O_3_ + DM group. ^#^*p* < 0.05, compared with the respective As_2_O_3_ group without PD98059.

*ERK signaling played a role in arsenic-inhibited osteoblastogenesis*. Because ERK phosphorylation has been shown to be involved in osteoblast differentiation (Ghosh-Choudhury et al. 2007; Lai et al. 2001), we investigated the effect of low-dose arsenic on ERK signaling during BMSC differentiation. Two As_2_O_3_ doses (0.5 and 1 μM) enhanced ERK phosphorylation during osteoblast differentiation; this enhancement was reversed by the ERK inhibitor PD98059 (Figure 3A,B). PD98059 also reversed As_2_O_3_-inhibited Runx2 protein expression (Figure 3B), ALP activity (Figure 3C), osteoblast mineralization (Figure 3D), and *Bmp2* (Figure 4A) and osteocalcin (Figure 4B) mRNA expression during osteoblast differentiation. PD98059 alone (10 or 20 μM) did not affect these osteoblastogenesis markers (Figures 3B–D, 4A–C). These results suggest that As_2_O_3_ inhibits osteoblast differentiation via an ERK-dependent signaling pathway.

**Figure 5:**
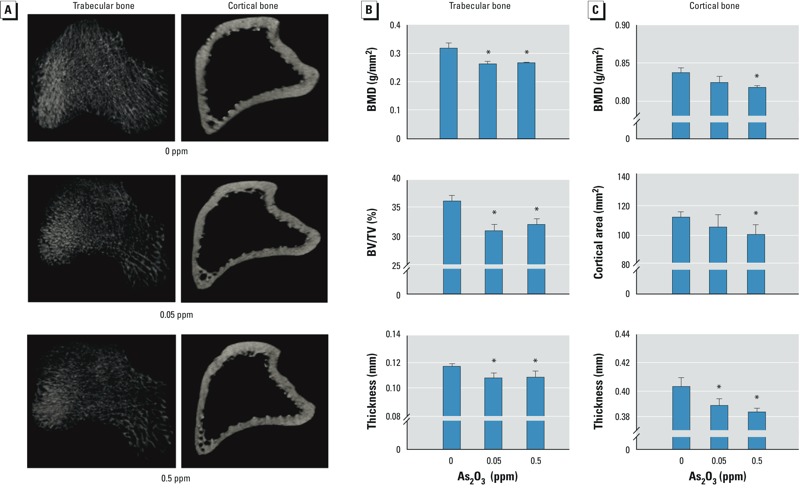
Long-term exposure to As_2_O_3_ decreased bone microstructure and BMD in rats. Rats were administered 0, 0.05, or 0.5 ppm As_2_O_3_ in drinking water for 12 weeks. (*A*) Representative photomicrographs of trabecular and cortical bone from the metaphysis and diaphysis regions of the proximal tibia as scanned by μCT. (*B,C*) Morphometric results for (*B*) trabecular bone (BMD, BV/TV, and thickness) and (*C*) cortical bone (BMD, cortical area, and thickness). Data are mean ± SD (*n *= 4 rats/group) of three independent experiments. *p < 0.05, compared with the 0‑ppm As_2_O_3_ group.

**Figure 6:**
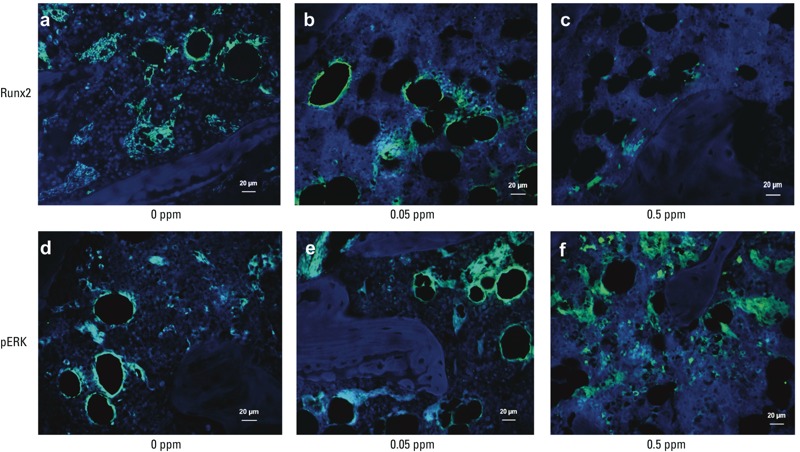
Immunofluorescent staining for Runx2 (top) and pERK (bottom) in bones of rats administered 0, 0.05, or 0.5 ppm As_2_O_3 _in drinking water for 12 weeks. Bars = 20 μm. Photomicrographs are representative of at least three independent experiments (4 rats/group).

*Arsenic altered bone microstructure and osteoblast differentiation in rats*. Twelve weeks after exposure to 0.05 or 0.5 ppm As_2_O_3_ in drinking water, body weights of rats were not significantly affected (control, 334.3 ± 21.5; 0.05 ppm, 339.9 ± 19.2; 0.5 ppm, 345.5 ± 5.0 g; *n* = 8/group). In As_2_O_3_-treated rats, microstructures in trabecular and cortical bone were altered (Figure 5A). In addition, BMD, BV/TV, and thickness of trabecular bone (Figure 5B), and BMD, cortical area, and thickness of cortical bone (Figure 5C) were significantly decreased. Immunofluorescence staining in bones from As_2_O_3_-treated rats displayed decreased staining for Runx2 and increased staining for phosphorylated ERK (Figure 6).

**Figure 7:**
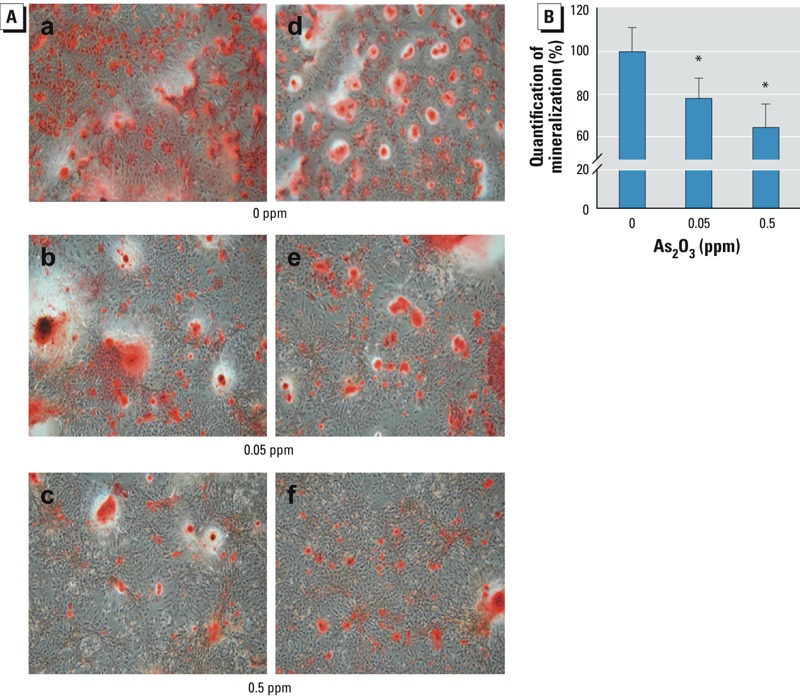
Osteoblast differentiation of BMSCs isolated from bones of rats administered 0, 0.05, or 0.5 ppm As_2_O_3_ in drinking water for 12 weeks. BMSCs were isolated from bones and cultured in differentiation medium for 20 days. (*A*) Photomicromicrographs and (*B*) quantitation of osteoblast mineralization. Data are mean ± SD (*n *= 4/group). *p < 0.05, compared with the the 0‑ppm As_2_O_3_ group.

In BMSCs isolated from bones of As_2_O_3_-treated rats, osteoblast differentiation (Figure 7A) and mineralization (Figure 7B) were significantly decreased, and ALP activity also significantly decreased (fold of control: 0.82 ± 0.09 in the 0.05-ppm group and 0.71 ± 0.08 in 0.5-ppm group; *n* = 4/group; *p* < 0.05). These results suggest that arsenic exposure caused the inhibition of osteoblast differentiation and altered bone microstructure and BMD in rats.

## Discussion

The main source of arsenic exposure in humans is arsenic-contaminated drinking water. Singh et al. (2007) estimated that arsenic concentrations in contaminated areas are several times higher than the maximum contamination level (the standard set by the World Health Organization and the U.S. Environmental Protection Agency) of 10 μg/L (0.01 ppm). Approximately 6 million people in West Bengal might be exposed to drinking water containing arsenic at > 50 μg/L (0.05 ppm) (Centeno et al. 2007). In an epidemiological study in Antofagasta, Chile, Borgono and Greiber (1971) observed that arsenic-related health problems resulted from exposure to contaminated drinking water, with arsenic concentrations as high as 800 μg/L (0.8 ppm). Arsenic has also associated with an increase in liver cancer mortality in both sexes, when arsenic levels are > 0.64 mg/L (0.64 ppm) (Lin et al. 2013). As_2_O_3_ has been reported to induce partial differentiation in acute promyelocytic leukemia cells at low concentrations (0.1–0.5 μM; about 0.02–0.1 ppm) but induce apoptosis at relatively high concentrations (0.5–2 μM; about 0.1–0.4 ppm) (Chen et al. 1997). Similarly, Yen et al. (2010, 2012) reported that low-dose As_2_O_3_ (0.1–0.5 μM; about 0.02–0.1 ppm) dose dependently inhibited *in vitro* skeletal muscle cell differentiation but higher concentrations (1–10 μM; about 0.2–2 ppm) induced apoptosis. In addition, Kesari et al. (2012) observed significant genetic damage in mice exposed to arsenic at the human equivalent reference dose (0.3 μg/kg/day), as well as its multiples (1.5–30 μg/kg/day). Obvious DNA damage was observed in bone marrow cells of mice exposed to arsenic (0.05 and 5 ppm) for 180 days (Singh et al. 2010). Exposure to 2.5–5 μM arsenite (about 0.5–1 ppm) could enhance the differentiation of preosteoclastic cells, suggesting that arsenic may result in increased bone resorption (Szymczyk et al. 2006). Rats treated with arsenite (0.21 mg/kg/day) for 45 days have also been found to have increased thickness of the growth cartilage and the hypertrophic zone, as well as trabeculae sealed to the cartilage (Odstrcil Adel et al. 2010). Recently, Hu et al. (2012) observed that, *in vitro*, relatively high concentrations of inorganic arsenic (≥ 2 μM; about 0.4 ppm) significantly decreased the differentiation of rat calvaria preosteoblasts; furthermore, they also found that short-term, high-dose arsenic (10 mg/kg/day for 4 weeks) administered by intraperitoneal injection, an unusual route of arsenic exposure, decreased both femur BMD and trabecular bone volume in rats. In the present study, we found that submicromolar As_2_O_3_ (0.5 and 1 μM) significantly reduced osteoblast differentiation of BMSCs *in vitro*. We also found that long-term exposure of rats to As_2_O_3_ in drinking water (0.05 and 0.5 ppm, 12 weeks)—doses found in human drinking water in arsenic-contaminated areas—significantly decreased BMD and bone Runx2 expression, increased bone ERK phosphorylation, and decreased osteoblast differentiation of BMSCs. These results suggest that exposure to arsenic at doses relevant to human exposure from drinking water may alter osteoblast differentiation of bone marrow cells and induce bone loss.

Arsenic can exist in the environment in several valency states (–3, 0, +3, and +5). It is mostly found in inorganic form as trivalent arsenite (As^3+^) and pentavalent arsenate (As^5+^) in natural water. The ratio of As^3+^/As^5+^ in water can greatly vary. In As-rich groundwater in Bangladesh, the ratios of As^3+^ to total arsenic range from about 0.1 to 0.9, but are typically around 0.5–0.6 (Jiang et al. 2013). In a previous study, Smedley and Kinniburgh (2002) found that the kinetic of oxygenation of As^3+^ is slow in the slightly acid range, around pH 5, and it is stable in the anoxic solution for up to 3 weeks. As_2_O_3_, a trivalent arsenic compound, can be released into air and water by natural or industrial processes, and it can form arsenite in alkaline solution. In the present study, to prevent or minimize oxidation of As_2_O_3_, we prepared the cell culture medium and the rats’ drinking water containing As_2_O_3_ every 2 and 3 days, respectively.

The ERK signaling pathway is involved in cell-matrix interactions in bone and the process of osteoblast differentiation (Ghosh-Choudhury et al. 2007; Lai et al. 2001; Wirries et al. 2013). Wu et al. (2012) suggested that osteoblastic differentiation of BMSCs is regulated by an ERK-related pathway. Exposure to arsenic has been reported to elevate ERK phosphorylation in various kinds of cells, such as endothelial cells (Wang et al. 2012), keratinocytes (Phillips et al. 2013), and neuronal mesencephalic cells (Felix et al. 2005), protecting against arsenic-induced damage. In contrast, a recent study found that sodium arsenite diminishes neuronal stem cell differentiation via overactivation of an ERK signaling pathway (Ivanov and Hei 2013). Activation of ERK signaling has also been shown to be involved in the inhibition of osteoblastic differentiation of vascular smooth muscle cells by ghrelin (Liang et al. 2012) or taurine (Liao et al. 2008). Tang et al. (2008) reported that PD98059 (20 μM) did not decrease ALP activity in rat osteoblasts. Lin et al. (2011) found that 20 μM PD98059 potentially induced rat preosteoblast differentiation, whereas Bai et al. (2013) reported that 10 μM PD98059 decreased osteoblast differentiation in rabbit BMSCs. In the present study, we found that As_2_O_3_ activated ERK activation during osteoblast differentiation of BMSCs and that PD98059 significantly reversed As_2_O_3_-inhibited osteoblast differentiation, suggesting that arsenic may inhibit osteoblastogenesis through an ERK-dependent signaling pathway. Taken together, these findings (Bai et al. 2013; Lin et al. 2011; Tang et al. 2008) and our results suggest that ERK activation can lead either to stimulation or inhibition of osteoblast differentiation pathways, depending on the system.

Runx2 is a master transcription factor that regulates bone formation and subsequently forms the fully functional osteoblasts (Lee et al. 2000). Celil and Campbell (2005) found that Runx2 activation is regulated by an ERK-dependent signaling pathway in human mesenchymal stem cells. Moreover, nuclear factor E2 p45-related factor 2 (Nrf2), a transcription factor for the regulation of many detoxifying and antioxidative genes, is known to be activated by ERK signaling (Cai et al. 2012; Khan et al. 2011). Hinoi et al. (2006) suggested that Nrf2 can negatively regulate osteoblast differentiation via an inhibition of the Runx2-dependent transcriptional activity (Hinoi et al. 2006). In the present study, we found that As_2_O_3_ activated ERK phosphorylation and inhibited Runx2 expression during osteoblast differentiation, which could be reversed by ERK inhibitor. The immunofluorescence co-localization of Runx2 and phosphorylated ERK has been shown in osteoblast cells (Li et al. 2010). The immunofluorescence staining for Runx2 and phosphorylated ERK in bones (Figure 6) might be mainly localized in osteoblast cells. This arsenic-activated ERK that down-regulated Runx2 expression during osteoblast differentiation of BMSCs may be through an ERK-activated Nrf2 signaling pathway. However, the role of Nrf2 in arsenic-inhibited osteoblast differentiation of BMSCs still needs to be clarified.

## Conclusions

In this study, we found that low-dose arsenic significantly reduced osteoblast differentiation of bone marrow cells *in vitro*. In rats, long-term exposure to arsenic in drinking water—at doses relevant to human exposure from drinking water—significantly altered bone microstructure and BMD. In addition, the up-regulation of ERK and the inhibitory effect of ERK inhibitor indicated that arsenic inhibited osteoblastogenesis through an ERK-dependent signaling pathway. Taken together, these *in vitro* and *in vivo* findings suggest that inorganic arsenic may be an environmental risk factor for osteoporosis.
